# The Present Status of Physiologic Therapy in Medical and Surgical Practice

**Published:** 1908-03

**Authors:** S. H. Monell

**Affiliations:** New York


// BUFFALO MEDICAL JOURNAL.

Vol. LXIII. MARCH, 1908. No. 8

### The Present Status of Physiologic Therapy in Medical and Surgical Practice

By S. H. MONELL, M. D., New York

PHYSICAL remedies as distinguished from drug remedies have no very clear place in the mental picture of the average prescriber, yet the modern practitioner is rightly expected to use all means at his command which will benefit his patient. Hence the writer is prompted to assume the task of briefly summing up the essentials required for a clinical understanding of the subject.

The aid of the underlying *vis medicatrix naturae*—the energies in nature acting upon the physiological functions of the body,—is accepted by the physician and surgeon and utilised. As they exist in diffused forms these natural energies constitute the primary physiologic therapy at our command. Modify them by scientific appliances and intensify their active dosage by artificial means, and we obtain therapeutic intensities of the same *vis mediacatrix naturae*. The machinist, inventor, and engineer, has done for these agents what the chemist has done for crude drugs in extracting alkaloids, and so forth. In both cases we are given instruments of greater precision and efficiency.

The investigations of physicists and clinicians have given most exact development to the world's knowledge of those chief groups classified as hydrotherapy, mechanotherapy, electrotherapy, phototherapy, and the actions of radiant and conducted heat. They have become an integral part of the materia medica, and conform to the same therapeutic principles of prescribing.

A modern equipment of physical remedies has two centuries of growth behind it, has been elaborated through the researches of many of the ablest men in medical history, and is the third arm of the profession. It has been difficult to select a satisfactory classifying name for this arm as a whole, and the term physiologic has crept into common usage. It is intended to signify that these agents exert their beneficial actions in harmony with the natural functions of the body, and tend to regulate them to normal when they deviate from it. By proper direction of dosage and administration, they are able to coöperate with the powers of nature inherent in the living tissues, and to increase or modify the actions which are necessary to restore the sound state of health. Within well understood limits and made to conform to well understood indications, the purpose in prescribing one of these agents is to make it do exactly the thing that nature would strive to do, to drive the disease out of the body.

Now, nature does not say, "try electricity on this person," or, "give this man a Scotch douche," or, "give that patient sparks," or, "give this woman a course of baths," or, "give that child vibration," yet in this way nearly all medical writings treat the matter of "physiologic" prescribing, and physicians talk methods instead of actions to meet indications. The subject is therefore confused and the clinical picture blurred in the medical mind. Methods cannot be prescribed for pathological states. Dosage is as requisite in a douche, a high frequency current, the application of light rays, a radiant heat bath or static spark, as it is in administering digitalis to energise the heart or a laxative to unload the bowels. The first thought of the physician after he selects his drug is the dose: the dose is the last thought of nearly all who employ a physiological remedy. Yet it is the controlling factor in all of them. Neglect of it makes abuse of the agent and explains complaints of failure.

Another common error is the custom of connecting together a given apparatus and a list of diseases which it is said to "be good for." This confuses the therapeutic principles and creates a fictitious viewpoint. The correct principle is to connect the remedy with the actions which it can be made to set up in the tissues, and in the mental picture of prescribing give these actions our first thought. In a given case select the actions needed, select the means of setting them up in the tissues, and direct these means,—the apparatus and methods,—so as to produce the prescribed effects. When this procedure is well carried out, we have rational physiologic therapy. When it is not carried out, we have abuses instead of therapeutics. The true basis therefore of prescribing "physiologic" agents is a physiologic action, or more than one, indicated by the pathologic state of the case. These actions are so different from the character of drug actions, that the diagnostic viewpoint must be different also. To the ordinary nosological diagnosis must be added a therapeutic diagnosis.

Take, for instance, an early case in which the practitioner halts between two opinions,—gout or chronic rheumatism. If it is gout his diet and medication will be one thing; if rheumatism, quite another. The importance of correct determination is obvious. This relates to drugs. But with physical remedies, we need solely to ascertain the character of the departure of the tissues from normal. If there is an inflammation, we note its extent and stage and decide what corrective action is indicated to allay it. If irritation exists, we consider what action upon the tissues will remove it. If a part is congested, we aim to regulate the circulation to normal. If there is pain, we ask what alteration of the tissues causes it, and what action upon the tissues will remove the cause. To combat the faulty metabolism, we aim to promote nutritional completeness by regulating the functions. No attempt is made to prescribe a physical agent for either gout or rheumatism, for physiologic remedies act on the state of the affected tissues. They thus support and supplement the chemical action of drugs, the two arms of medicine,—the internal and external,—making the treatment complete.

Again, take a case of suspicious signs in the chest. It does not readily clear up. There is no sputum with bacilli to confirm a nosological diagnosis. The internist here meets difficulties which often pave the way to regret. But the aid of the second arm of medicine is decisive. The prescription is based on the therapeutic diagnosis, the actual clinical indications of the tissues. Possibly some may find it difficult to forego the accustomed mental attitude of the internal prescriber, sufficiently to at once grasp the simple and equally rational attitude of the prescriber of physiologic remedies, but in practice both clinical pictures are equally clear to the student of both arms of medicine. No man objects to his left hand because he makes most use of his right, and in practice we employ the internal and external arms of medicines in perfect harmony, to assist the recuperative powers of nature.

And now, for a moment, let us study the simplicity and beautiful accuracy of this rational prescribing of agents, which we can direct with almost an approach to the definiteness of a surgical procedure. We are all aware of the long list of diseases discussed in standard textbooks, many of them being self-limited or curable. Admitting the inherent effort of nature to cure all really curable diseases herself, and knowing that she often succeeds, how does she do it? Not by reason of many antidotes for many diseases, but by trying to restore the normal rate of altered functions of affected tissues, for under ordinary conditions health is the proper working of a few physiological processes; mainly a half dozen. When the efforts of nature, or medical efforts joined to hers, succeed, the state of health is resumed, no matter what the disease may have been called.

Whether the condition can be restored to normal or not will depend upon the energy of the cause which disturbs the balance of the physiologic processes, but whether the cause is a germ, a chemical agent, or traumatism, the principles of physiologic action are the same. If the attack of the given enemy is too strong for nature to resist, the patient will succumb unless we help her. In bestowing this aid so far as it can be afforded by physical agencies, the main actions that are required in the treatment of disease and post-operative conditions are briefly as follows:

An increase or decrease of the blood supply of a part of the body—the part affected.

An increase or decrease in the rate or energy of the heart or regulation of its action.

An increase or decrease in the processes of secretory cells.

An increase or decrease in excretory processes.

An increase or decrease in the rate or extent of the oxidation of food.

An increase or decrease in the contractile function of muscle fibers.

Regulation of the nerve centers to normal action.

And so on to the end of the physiological necessities of life and health.

These are the things that are behind the majority of the symptoms and sufferings of chronic diseases. In the treatment of such cases, in both curable and incurable diseases, the patient will feel well in proportion to the completeness with which the functional processes of the body can be restored to normal, even when the disease is organic. Physiologic therapy has attained its high place in modern medicine solely for the reason that by means of competent apparatus and proper methods of dosing the amount, rate, force, quality, extent and character of the selected agents, the physician can make these intensified energies of nature increase, decrease, or otherwise modify, each and every physiological process that is within the reach of human skill and influence.

For instance, by one means of local action we can increase the supply of good blood in a part and improve its nutrition by thus feeding it. By opposite means we can lessen the blood supply of local tissues and thus reduce congestion and abate pain. By one method we can produce gentle or energetic contractions of muscle fibers and thus stimulate them to greater strength; by another method we can reduce irregular and spasmodic contractions and thus relieve cramp and nervous irritation. By one method we can increase the amount of secretion of a gland and stimulate it when it falls below normal; by another method we can lessen secretion when it is excessive, as we dry up a fluent coryza or effectually banish hay fever.

In far reaching importance the function of muscle contraction is almost coextensive with life; it does the work of the heart, it carries on the arterial circulation directly and the venous circulation indirectly; it performs the mechanics of respiration; it is the origin of every move we make; it is the sustaining factor in nutrition and strength and is vital to living tissues. And the only means by which the physician can set up tonic, energising, refreshing, nutrition-promoting contractions in any muscle apart from reflex action or volition is through the aid of the greatest of all physical agents—namely, electricity. Were no other medical work possible with electricity this command over the function of muscle contraction would alone make it indispensable.

Furthermore, consider the great variety of patients who need local or general elimination of toxic products of one kind or another. Very exact laboratory tests have proved the efficiency of the modern sweat bath of radiant heat, to which the human tissues are practically porous. To a physical agent—light—we are indebted for the most useful supplement to other depurative measures that we possess. The importance of its elimination by the skin is sufficiently great, but it also increases the subsequent elimination through the excretory internal organs, acting at the same time as an alterative tonic.

This fragmentary list of actions may not at first prompt the surgeon to realise in how many conditions both before and after operation he can obtain aid from these agents. At first reading not every physician will be able to see just how these actions fit into his treatment of gout, rheumatism, diabetes; of cardiac, renal, pulmonary and nervous affections; of debilities, of lessened tissue resistance, and of practically almost the whole range of chronic illhealth, especially in those conditions in which the limitations of internal medicine leave much to be desired.

In a recent article Dr. J. E. Winters quotes Dr. Andrew H. Smith as saying that since the introduction of salicylic acid, he experienced no difficulty in getting his patients about three-fourths well. If some one would tell him how to cure the remaining fourth of their diseases he would be greatly obliged. The way to "cure the remaining fourth of their disease" is most likely to be found in physiologic therapy. Its resources will at least complete the chain of treatment and secure all the improvement that is medically possible.

This sufficiently indicates the present status of this branch of medicine. Further evidence of advance is to be found in the increased attention paid by manufacturers to the improvement of apparatus, and in the increasing recognition of these agencies by the profession. No well-equipped hospital or sanitarium would now try to get along without some selection from their resources. The main deterrent to widespread use in private practice in the treatment of chronic diseases is primarily the cost, space required, and need of technical skill, rather than any longer dispute of their place in therapeutics. Some of these impediments will be modified by still further inventions and the third arm of the practitioner will be deemed as indispensable by the profession as the older and better taught procedures. Neither the surgeon nor physician will consent to deprive his patients of the help of these external allies after experience has demonstrated their value.

230 West One Hundred and Seventh Street.

